# Localization and Expression of Aquaporin 0 (AQP0/MIP) in the Tissues of the Spiny Dogfish (*Squalus acanthias*)

**DOI:** 10.3390/ijms27031317

**Published:** 2026-01-28

**Authors:** Christopher P. Cutler, Casi R. Curry, Fallon S. Hall, Tolulope Ojo

**Affiliations:** 1 Department of Biology, Georgia Southern University, Statesboro, GA 30460, USA; 2 Department of Biology, Baylor University, Waco, TX 76706, USA

**Keywords:** aquaporin 0, major intrinsic protein, spiny dogfish, osmoregulation, localization

## Abstract

The aquaporin 0 (*AQP0*)/major intrinsic protein of eye lens (MIP) cDNA was cloned and sequenced. Initial studies of the tissue distribution of mRNA expression proved to be incorrect. Subsequent experiments showed that *AQP0* mRNA is expressed strongly in the eye with moderately strong expression in the kidneys and some expression was seen in the brain and muscle tissue, and very low expression in the esophagus/fundic stomach. Another set of PCR reactions with five times the amount of cDNA additionally showed mRNA/cDNA expression in the liver, rectal gland, and a very low level in the intestine. Sporadic expression of different pieces of *AQP0* cDNA was seen in various experiments in gill and pyloric stomach. A custom polyclonal antibody was produced against a region near the C-terminal end of the AQP0 protein sequence. The antibody gave a band of around the correct size (for the AQP0 protein) on the Western blot, which also showed a few other higher-molecular-weight bands. The antibody was also used in immunohistochemistry, and in the kidney, it showed staining in the proximal II (PII), intermediate segment I (IS I), and late distal tubule (LDT) parts of the sinus zone region of nephrons as well as some staining in the bundle zone tubule segments, suggesting a role for AQP0 as a water channel. In the rectal gland, the antibody showed weak apical membrane staining in a few secretory tubules near the duct, but also somewhat stronger staining in cells appearing to connect various secretory tubules, suggesting a role in cell–cell adhesion. In the spiral valve intestine side wall and valve flap, after signal amplification, weak antibody staining was seen in the apical and lateral membranes of epithelial cells adjacent to the luminal surface. There was also some staining in the intestinal muscle. In the rectum/colon, staining was seen in a layer of cells underlying the epithelium and in some muscle layers. In the gill, there was very weak staining in secondary lamellae epithelial cells and in connective tissue surrounding blood vessels and blood sinuses. The low level of transcript expression in the rectal gland, gill, and intestinal tissues suggests caution in the interpretation of the immunohistochemical staining in these tissues.

## 1. Introduction

Aquaporin 0 (*AQP0*) or MIP (major intrinsic protein) was the first member of the aquaporin gene family to be discovered [1]. Peter Agre was awarded the Nobel Prize for Chemistry in 2003 for discovering *AQP1* in 1991 [2], but there was a prior family member (homolog) in the gene bank when *AQP1* was deposited. That family member was MIP, which was then also named *AQP0* as it pre-dated AQP1.

The AQP gene family in mammals consists of thirteen members, *AQP0-12* [3], with three main sub-groups: water-selective *AQP*s (*AQP*s *0*, *1*, *2*, *4*, *5*, *6*), which predominantly transport water; aquaglyceroporins (*AQP*s *3*, *7*, *9*, *10*), which transport small solutes such as urea and glycerol; and more divergent *AQP*s (*8*, *11*, *12*). In wider vertebrates, there are also four more *AQP*s that exist, *AQP13-16* [4].

*AQP0* in mammals is found almost exclusively in the lens of the eye [3], although some expression has also been shown in rat liver and horse testes [5,6,7,8]. AQP0 has a relatively low but significant water permeability (around 43× lower than AQP1, [9,10]), and as well as monomers of AQP0, it predominantly forms tetramers and even orthogonal arrays of tetramers in the lens [11,12,13]. In addition to being a channel, AQP0’s functions include the formation of adhesive junctions between lens fiber cells with cell–cell interactions involving either AQP0-AQP0 or AQP0 negatively charged lipids (phosphatidylserine) in opposing cell membranes [8,14,15,16]. Evidence suggests that AQP0 tetramers are post-translationally cleaved at both the N and C terminal ends before forming cell–cell junctions [11,12,13,17,18]. Junctional AQP0 complexes are thought to lose their water permeability [13,18]. AQP0 has also been shown to possess a voltage-dependent unselective ion conductance that preferentially transports anions (chloride), as well as being able to transport sucrose and glycerol [11,19]. The ion conductance was shown to be gated and was regulated by calcium, calmodulin, pH, and by phosphorylation of the AQP0 C-terminal tail [13,19,20,21]. For reviews on AQP0 in mammals, see [8,12,13,16,21,22,23,24,25,26,27,28].

In teleost fish, there are often two copies of the *AQP0* gene due to a genome duplication event, and these duplicates show sub-functionalization but are both required for normal lens function [8]. There is also a slightly different distribution of *AQP0* duplicate expression compared to mammals, where, as well as *AQP0* copies being expressed in the eye, *AQP0a* is expressed in the testes [29] and *AQP0b* is expressed in the ovary [30]. Unlike in mammals, both of these teleost duplicate paralogs have been shown to possess water permeabilities of a similar magnitude or only slightly lower than that of other AQPs from the same species [29,30]. In salmonids, such as Atlantic salmon (*Salmo salar*, which have an additional genome duplication in comparison to most other teleosts), there are four copies of the *AQP0* gene (*0a1*, *0a2*, *0b1*, *0b2*). All four copies, as well as being expressed in the lens of the eye, ovary, and testes, are expressed at very low levels in a number of other tissues such as gills, kidney, and intestine/rectum [31]. The four salmon AQP0s, as in mammals, are all thought to have cell–cell adhesion capabilities similar to human AQP0 [32]. Both zebrafish (*Danio rario*) and salmonid *AQP0* paralogs’ water permeability characteristics display some sensitivity to different pH values, except for those of salmon AQP0b2 [31]. A sensitivity to different pH levels has also previously been shown for teleost AQP3b [33]. For reviews concerning teleost AQP0 and other AQPs, see [29,34,35,36,37].

This project, investigating spiny dogfish (*Squalus acanthias*) AQP0, came about partly through serendipity. Elasmobranchs such as the spiny dogfish shark are now known to have thirteen *AQP*s, including *AQP0* [4,38,39]. But this was unknown when students in a 2010 undergraduate lab class, replicating degenerate PCR experiments using primers from a study concerning spiny dogfish *AQP4* [40], unexpectedly amplified and cloned a cDNA fragment from kidney cDNA that, when sequenced, transpired to be a partial copy of *AQP0* cDNA. The complete AQP0 cDNA sequence was then acquired using Smarter RACE PCR using kidney cDNA, and a custom-made affinity-purified anti-AQP0 peptide-antigen-based rabbit polyclonal antibody was made against a short peptide sequence from the translated cDNA sequence. The identification of *AQP0* using kidney cDNA was unexpected and initial PCR amplifications of the exon 2 region, using Intron-Exon-Junction (IEJ)-located primers, showed *AQP0* expression occurred in every tissue of the spiny dogfish studied, including the kidney [34]. While that still seems to be the case, the situation regarding the presence of *AQP0* transcripts in some tissues is now more complex.

The kidney of sharks is more complex than mammalian kidneys, with effectively an extra loop. Nephrons move between two zones, the sinus zone (tubules interspersed with blood sinus spaces) and the bundle zone (bundles of five tubules enclosed within a bundle sheath). Tubules start at the glomerulus at the edge of the sinus zone and then the neck segment is the first bundle zone tubule; the tubule forms a loop (becoming proximal Ia tubule; PIa) and returns to the sinus zone. The first sinus zone tubule loop starts with proximal Ib (PIb), followed by proximal II (PII) and intermediate I (IS I) segments. The nephron then heads back into a second bundle zone loop with intermediate II (IS II) and early distal tubule (EDT) segments. The second loop then heads back into the sinus zone for its second loop, which is the late distal tubule (LDT). The nephron then enters the bundle zone becoming the collecting tubule (CT) before becoming the collecting duct (CD). For schematic diagrams of this nephron structure, see [41].

This study was carried out to determine the possible roles that AQP0 might be playing in various (particularly osmoregulatory) tissues. As AQP0 is a member of the aquaporin water channel family and has been shown to exhibit water permeability properties in some teleost fish species, a role for AQP0 in osmoregulatory processes was thought to be likely, but this was fundamentally a discovery science study rather than a hypothesis-driven one, attempting to acquire basic information about the expression of the *AQP0* gene and its protein for the first time in elasmobranchs.

## 2. Results

**Figure 1:**
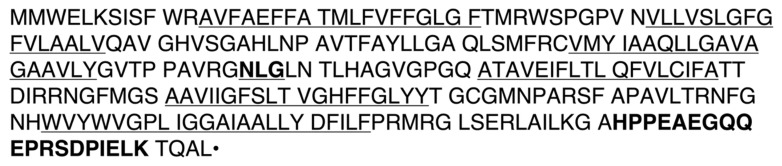
Amino acid sequence of spiny dogfish AQP0 (single letter codes). The putative membrane-spanning regions are underlined. A potential unorthodox N-glycosylation site is underlined and bolded. The region of the sequence used to make an AQP0 peptide-antigen-based affinity-purified rabbit polyclonal antibody is bolded. The • represents the stop codon. The encoded sequence would produce a protein of 28.4 kDa without any N-glycosylation or other post-translational modifications.

The sequence of spiny dogfish *AQP0* was determined using degenerate and RACE PCR, and the sequence (Ac. No. PX789079) is now available in the genebank. The derived amino acid sequence of dogfish AQP0 was 97% homologous to that from the evolutionarily divergent catshark (*Scyliorhinus canicula*; Ac. No XM_038786460) and great white shark (*Carcharodon carcharias*; Ac. No. XM_041180161), showing a high level of conservation of AQP0 sequences amongst sharks. The level of homology of the dogfish AQP1 amino acid sequence (to AQP0), which was similarly derived from AQP4 by duplication, is 51.7% [4,42]. The C-terminal end region used to make an AQP0 anti-peptide antibody (see Figure 1) showed a low level of amino acid homology (26%) in comparison to AQP1.

During further experiments to perform quantitative PCR, the primers, previously used for the original RACE and tissue PCR experiments, which were located at the IEJ of exon 2 of the *AQP0* gene, unexpectedly failed to give clean amplification, and an RT-minus reaction (using total RNA) showed amplification from contaminating genomic DNA. This was probably due to too much exon 2 sequence on the 3′ ends of the primers. This led to a series of further PCR experiments to clarify the mRNA expression profile of *AQP0* in dogfish tissues and to generate other more specific primers for quantitative PCR.

### 2.1. Tissue Distribution and RACE PCRs

**Figure 2:**
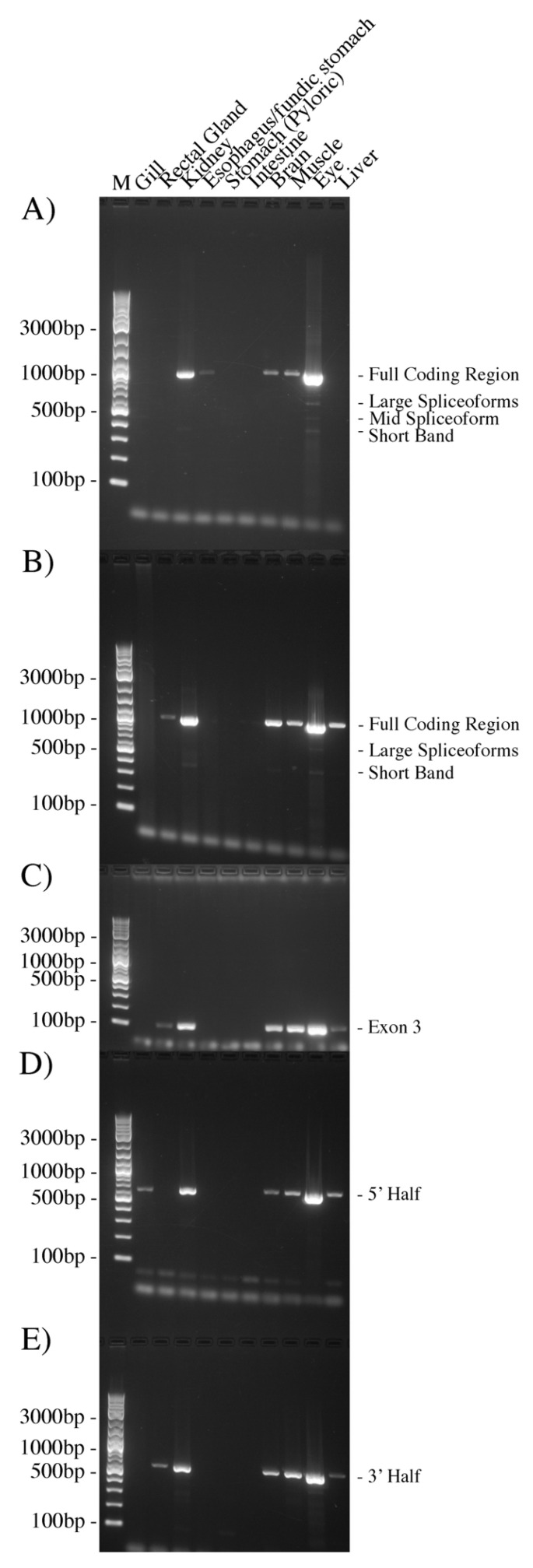
RT-PCR of different parts of the *AQP0* from different dogfish tissue cDNAs. (**A**) PCR reactions (1 µL of cDNA per reaction) amplifying the whole coding region of the *AQP0* cDNA. Three additional smaller bands were generated in the eye cDNA reactions (as indicated). (**B**) PCR reactions (5 µL of cDNA per reaction) amplifying the whole coding region of the *AQP0* cDNA. (**C**) PCR amplification of the region containing *AQP0* exon 3 using IEJ-based primers (which overlap with exons 2 and 4, respectively). (**D**) Amplification of the 5′ half of the *AQP0* cDNA coding region. (**E**) Amplification of the 3′ half of the *AQP0* cDNA coding region. Samples in (**A**,**B**,**D**,**E**) were separated using a 1.2% agarose gel. Samples in C used a 1.5% agarose gel. The DNA marker ladder (M) was 2-log ladder (NEB).

Initial RT-PCR experiments amplified the full coding region (920 bp) of *AQP0* (see Figure 2A). Transcripts were amplified in the kidney, esophagus/fundic stomach, brain, muscle, and eye. At least three additional bands were seen in the eye reactions. These were cloned and sequenced. The larger band (large spliceoforms) contained two similar-sized products. The first (*AQP0-SV1*; Ac. No. PX789078) was missing 327 bp or 109 amino acids of the sequence from the alanine amino acid at position twelve of the sequence to the last amino acid of exon 1, the threonine at position 121 of the sequence (inclusive). The second product (*AQP0-SV2*; Ac. No. PX789080) was missing 313 bp from the leucine amino acid at position 44 of the sequence (inclusive), but then the omission caused a frame shift in the reading frame of the sequence. The mid spliceoform band also had two products. The first, (*AQP0-SV3*; Ac. No. PX789081) was missing 444 bp/148 amino acids from the alanine at position 92 of the sequence (in exon 1) to the glycine at position 240 (in exon 4). The second mid spliceoform version (*AQP0-SV4*; Ac. No. PX910923) had only the first four amino acids of the AQP0 protein sequence (13 bp) and then 446 bp missing, leading to a frameshifted sequence, which could potentially produce a 47 amino acid protein. The smallest band on the gel (short band) was curious in that it was the end 387 bp piece of AQP0, including the antisense primer on its far end, but with no sense primer at the beginning of the sequence. It is unclear how this product was generated in the PCR. In order to improve on these results and in light of the other sets of PCR results (below), it was decided to repeat the amplifications of the full coding region but using 5 µL of cDNA rather than 1 µL (as in Figure 2A; see Figure 2B). Compared to the original amplifications, this additionally resulted in relatively strong amplification in the liver with a moderate band in the rectal gland and a faint band in the intestine. There was, however, only the merest hint of a band in the esophagus/fundic stomach. Still no bands were seen in the gill or pyloric stomach.

New primers (Squal AQP0 QPCR sen and Squal AQP0 QPCR anti) were generated for quantitative PCR based on the IEJs at either end of exon 3. This generated a 115 bp product. A pair of extended versions of these primers (Squal AQP0 QPCR senXL and Squal AQP0 QPCR antiXL) was also produced for regular RT-PCR. Tissue RT-PCRs with the extended primers (see Figure 2C) gave amplification products in the rectal gland and liver but not in the esophagus in comparison to the amplifications of the full-length coding region (in Figure 2A). Due to the apparent lack of expression in the gill, further amplifications of each (overlapping) half of the *AQP0* sequence were performed (5′ half primers: Squal AQP0 CDS sense and Squal AQP0 QPCR antiXL, product = 648 bp; 3′ half).

Primers: Squalus AQP0 3′R-QPCR Sen 2 and Squal AQP0 CDS anti, product = 544 bp). In PCR reactions using these primers (see Figure 2D), the 5′ half was amplified in the gill and liver but not in the esophagus in comparison to the amplifications of the full-length coding region (in Figure 2A). The 3′ half (Figure 2E) was amplified in the rectal gland and liver but not in the esophagus in comparison to the amplifications of the full-length coding region (in Figure 2A).

**Figure 3:**
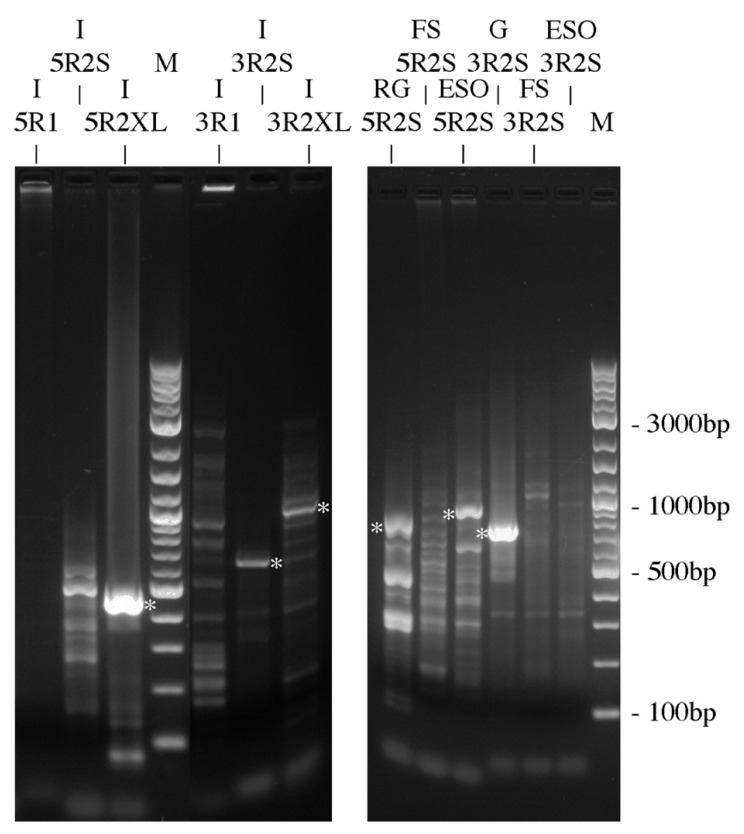
Results from two different gels of 5′ and 3′ RACE RT-PCR reactions from various tissue cDNAs on 1.2% agarose electrophoresis gels. The labels at the top are in two layers, with a | symbol indicating the gel well/track. I = intestine, RG = rectal gland, FS = fundic stomach, ESO = esophagus, and G = gill. 5R1 and 3R1 are first-round PCR reactions and 5R2 and 3R2 are second-round nested PCR reactions. The S indicates the Smarter kit’s short nested primer. XL indicates a longer version of the nested primer. M is the 2-log ladder DNA marker (NEB). White asterisks indicate the bands that were cloned and sequenced. The *AQP0* GSP primers used were the Squalus AQP0 5′R-QPCR Anti remake (5′ RACE; for R1) and Squalus AQP0 3′R-QPCR Sen remake (3′ RACE; for R1) and the Squal AQP0 5 Race 2 (5′ RACE; for R2) and Squal AQP0 QPCR senXL primers (3′ RACE; for R2).

**Figure 4:**
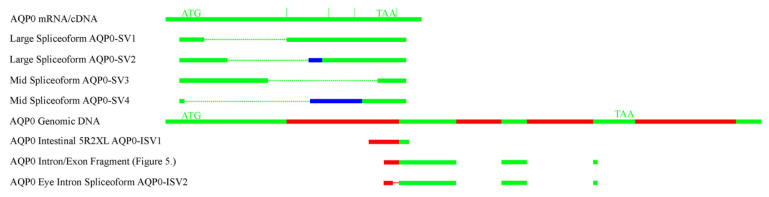
A schematic line diagram of the various *AQP0* spliceoforms and fragments (not to scale but with some adjustments for different sizes). Green lines represent the five exons, red lines represent the four introns, and blue lines represent the region coding for protein after a frameshift in the sequences, which also end at a new premature stop codon. Dotted lines represent regions missing from the spliceoforms. On the cDNA/mRNA diagram, vertical lines represent the IEJs of the five exons. Gaps between exons are present because a cDNA segment was amplified which did not have the introns (which are absent from a cDNA/mature mRNA version) at those locations. The start codon (ATG) and stop codon (TAA) are indicated.

**Figure 5:**
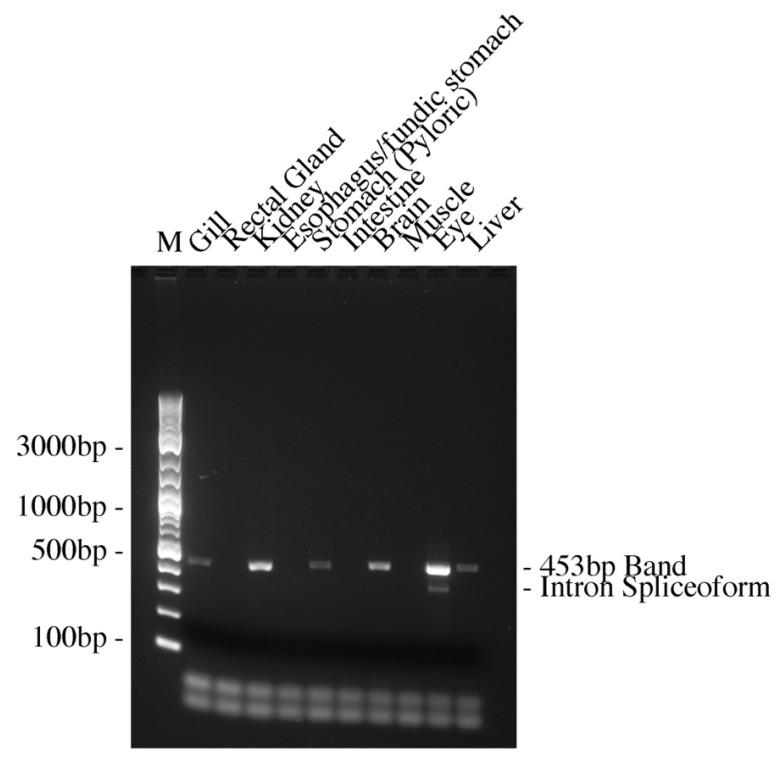
Amplifications of an intron 1 to the end of exon 3 *AQP0* fragment using RT-PCR and the various tissue cDNAs listed and run on a 1.5% agarose electrophoresis gel. The amplifications were to test whether an intestinal 5′RACE fragment identified was a cDNA or genomic amplification product. The cDNA fragment was 453 bp, whereas the genomic version would contain an additional intron 2 segment adding around 666 bp (based on whitespotted bambooshark sequence). A smaller splice variant band was seen in the eye cDNA track (Intron Spliceoform; AQP0-ISV2).

To investigate these results, some further nested 5′ and 3′ Race RT-PCR reactions were performed (see Figure 3). The 5′ RACE products cloned and sequenced from the rectal gland and esophagus and the smaller intestinal 3′ RACE product produced with the Smarter RACE kit nested short primer (3R2S) were all unrelated to *AQP0*. The 3′RACE products from the gill and intestine were *AQP0* products. The intestinal 5′ RACE product (AQP0-ISV1; 5R2XL; Ac. No. PX789082; see Figure 4. for a schematic diagram of the fragment and all spliceoforms) was a piece of *AQP0* that contained the *AQP0* coding region on its 3′ end but with an unrelated sequence (presumed to be intron 1) at its start (5′ end). As the sequence extended essentially from intron 1 into exon 2, the product could be either a genomic amplicon or a cDNA transcript. So a primer was made within the intron 1 sequence (Squal AQP0 5′ intron sen 2) and used with the Squal AQP0 QPCR antiXL primer located at the far end of exon 3 (IEJ). Hence, the product produced would be 453 bp from a cDNA transcript but far bigger than the genomic DNA version, as it would also contain intron 2 (666 bp in the whitespotted bambooshark *AQP0* gene). The PCR reactions performed (see Figure 5) interestingly gave products of the expected size of cDNA transcripts in the gill, kidney, pyloric stomach, brain, eye, and liver and no genomic versions. Just to check for the presence and size of intron 2, primers were made to amplify the region containing it, and a band of the expected size (based on whitespotted bambooshark sequence) was obtained from an RT-minus total RNA sample containing genomic DNA. Additionally, in the eye RT-PCR reaction (Figure 5), a smaller band was obtained (intron spliceoform; see Figure 5) and this was also cloned and sequenced (*AQP0-ISV2*; Ac. No. PX789083). The sequence had the (3′) end 95 bp of the intron 1 part of the fragment sequence missing in comparison to the originally obtained partial sequence of intron 1.

### 2.2. Quantitative PCR

**Figure 6:**
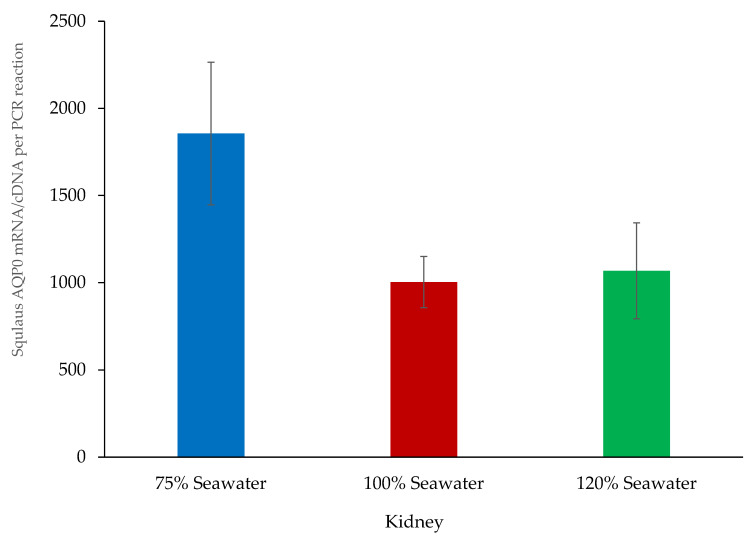
Quantitative PCR graph of the number of cDNA molecules per PCR reaction in dogfish kidney samples from fish acclimated to 75% SW, 100% SW, and 120% SW, as indicated. There were no statistically significant differences (ANOVA with Fisher’s PLSD post hoc testing; see method section) between groups. *N* = 6 fish/RNA/cDNA samples per group.

In the kidney, the IEJ primers that amplify exon 3 (Squal AQP0 QPCR sen and Squal AQP0 QPCR anti primers) were used to carry out quantitative PCR using a set of total RNA samples/cDNAs from the kidneys of dogfish acclimated to different environmental salinities. The results (see Figure 6) outlined that the dogfish that were acclimated to 75% seawater (SW) showed somewhat higher average levels of mRNA expression than fish acclimated to either SW (100%) or 120% SW, although this higher level was not quite statistically significant (*p* = 0.0606 and 0.0805, respectively).

### 2.3. Western Blotting

**Figure 7:**
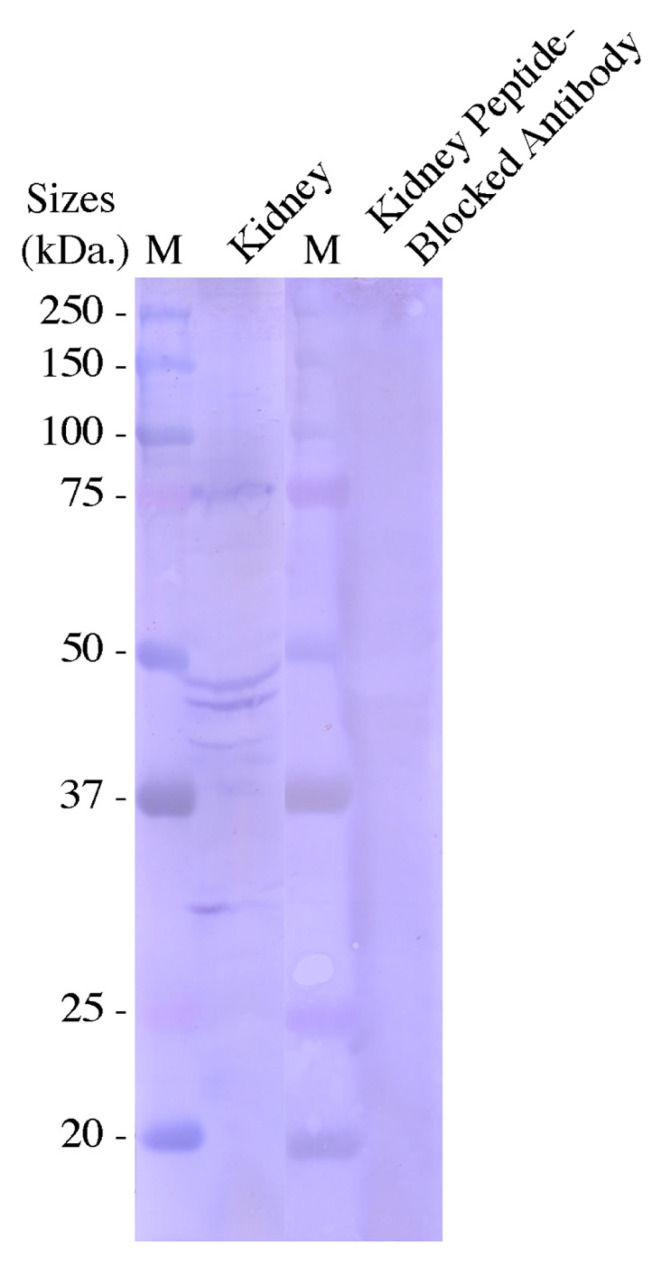
Western blots using the rabbit anti-dogfish AQP0 peptide affinity-purified polyclonal antibody. The protein sample used was 300 µg of purified plasma membrane protein run on a 10% Laemmli polyacrylamide gel and electroblotted onto PVDF membrane. The two filters were incubated with antibody (1 in 400 dilution) or peptide-blocked antibody for 1 h at room temperature and donkey anti-rabbit IgG with alkaline phosphatase attached for 1 h. The filters were incubated in NBT/BCIP alkaline phosphatase substrate for 3 min. Antibody blocking was carried out for >1 h using 50 µg/mL peptide antigen. Protein marker (M) is Precision Plus Kaleidoscope protein markers (Biorad, Santa Rosa, CA, USA).

As mentioned above in Figure 1, a region near the C-terminal end of AQP0 was used to make a rabbit anti-dogfish AQP0 peptide affinity-purified polyclonal antibody for use in Western blotting and immunohistochemistry. On the Western blot, using a kidney-purified membrane protein sample (Figure 7), a band of around 30 kDa was seen, as well as bands at around 43, 46, 49, and 75 kDa. These bands were blocked out in the blot incubated in AQP0 antibody pre-blocked with the peptide antigen.

### 2.4. Immunohistochemistry

**Figure 8:**
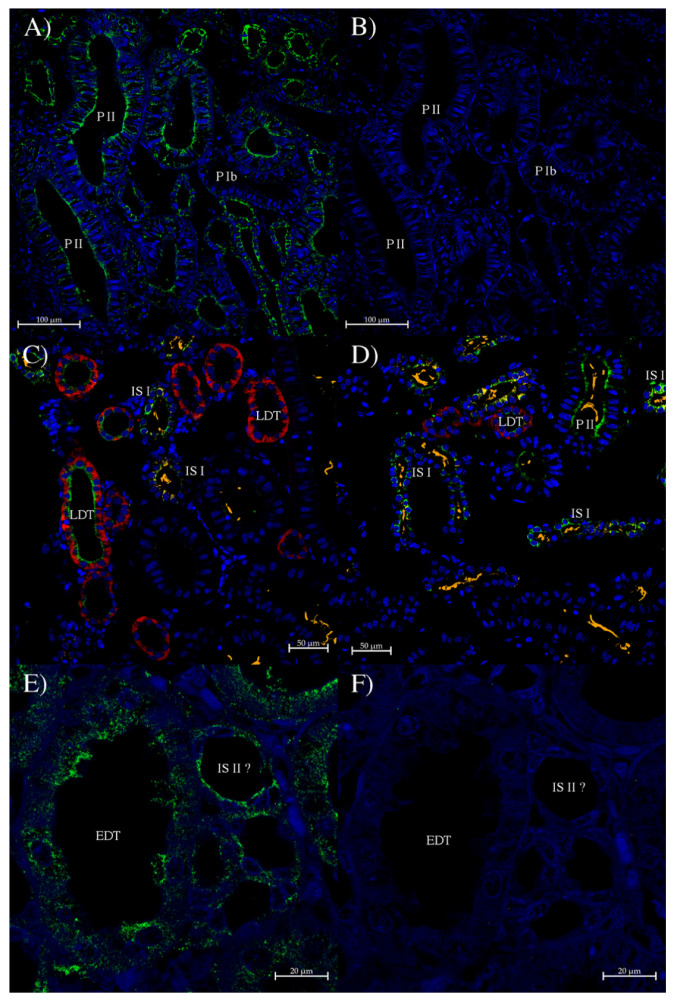
Immunohistochemistry of dogfish kidney using the AQP0 polyclonal antibody. Sections (**A**–**D**) are of the sinus zone and (**E**,**F**) are of the bundle zone. (**A**,**E**) were incubated with the rabbit AQP0 antibody and an Alexa 488 plus donkey anti-rabbit IgG secondary antibody (green; Thermofisher, Carlsbad, CA, USA). (**B**,**F**) are control serial sections (compared to (**A**,**E**) respectively) incubated with peptide antigen-blocked AQP0 antibody and an Alexa 488 plus donkey anti-rabbit IgG secondary antibody (green stain; Thermofisher, Carlsbad, CA, USA). (**C**,**D**) were generated with a tyramide amplification kit using Alexa 488 (AQP0 antibody, green stain; Thermofisher, Carlsbad, CA, USA). They were also incubated with either AQP4/2 antibody (**C**) or AQP3 antibody (**D**), which were directly labeled with a CF633 dye attached using a mix-n-stain kit (red stain; Biotium, Fremont, CA, USA), which strongly label different parts of LDT tubules. There was additional incubation with mouse anti-acetylated tubulin antibody (Sigma, St. Louis, MO, USA), detected with highly cross-absorbed goat anti-mouse IgG secondary antibody with Alexa 555 dye attached (orange), which labels cilia in the first sinus zone loop (PIb, PII, IS I). Images were acquired using a Zeiss LSM 710 laser-scanning confocal microscope (Zeiss, Oberkochen, Germany). (**A**,**B**) were acquired using a 20× lens and zoom setting of 0.8. (**C**,**D**) were acquired using a 20× lens and zoom setting of 1.0. (**E**,**F**) were acquired using a 40× lens and zoom setting of 1.7. Scale bars show relative scaling. All sections had autofluorescence quenched using a True Black plus autofluorescence quencher (Biotium). Blue staining is from DAPI nuclear counterstain present in the Prolong Diamond coverslip mounting medium (Thermofisher, Carlsbad, CA, USA).

The AQP0 antibody was also used in immunohistochemistry experiments in osmoregulatory tissues. In kidney, staining was seen in both the sinus zone and bundle zone tubule segments (Figure 8).

The AQP0 antibody in the kidney showed patchy staining in most tubule segments. In the sinus zone, stain was seen in some but not all PII and IS I tubules. In the LDT segment, some tubules at the beginning of the LDT (strong AQP4/2 staining) had apical membrane AQP0 antibody staining but others did not. There was also some lower-level intensity AQP0 antibody staining in some AQP3-expressing tubules near the end of the LDT. Also, in bundle zone tubules, there was strong but patchy AQP0 antibody staining in the IS II/EDT loop, with lower and more punctate staining in the other three tubules (Neck, PIa, and CT).

**Figure 9:**
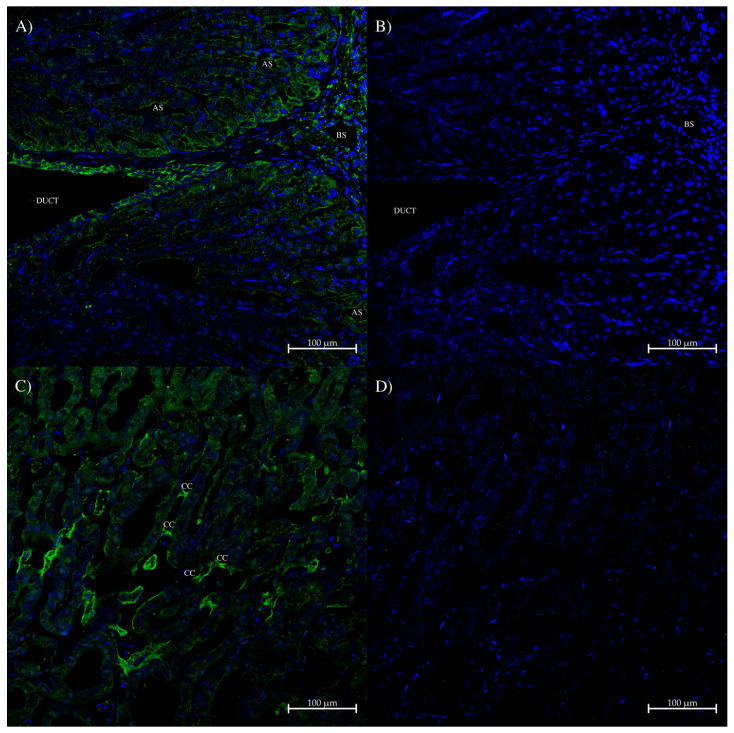
Immunohistochemistry of dogfish rectal gland using the AQP0 polyclonal antibody. (**A**,**C**) were incubated with the rabbit AQP0 antibody and an Alexa 488 donkey anti-rabbit IgG secondary antibody (green; Thermofisher, Carlsbad, CA, USA). (**B**,**D**) are control serial sections (compared to (**A**) respectively) incubated with peptide antigen-blocked AQP0 antibody and an Alexa 488 plus donkey anti-rabbit IgG secondary antibody (green stain; Thermofisher, Carlsbad, CA, USA). Labeling is AS = apical membrane staining; CC = connective tissue cell staining; BS = blood sinus; DUCT = central duct of the gland. Images were acquired using a Zeiss LSM 710 laser-scanning confocal microscope (Zeiss, Oberkochen, Germany). All panels were acquired using a 20× lens with a zoom level of 0.8 (scale bars as indicated). All sections had autofluorescence quenched using a True Black plus autofluorescence quencher (Biotium). Blue staining is from DAPI nuclear counterstain present in the Prolong Diamond coverslip mounting medium (Thermofisher, Carlsbad, CA, USA).

In the dogfish rectal gland, there was a small amount of AQP0 antibody staining of the apical membrane of rectal gland tubule cells that occurred mainly towards the central duct of the gland (Figure 9A). There was also some staining in the layer of cells lining the duct (Figure 9A). Lastly, in patches near the duct, there was some more intense AQP0 antibody staining in connective tissue cells that appeared to be associated with adhering one secretory tubule to another (Figure 9C). In some places, these connections appeared to be single connective cells; in other places, there were multiple cells involved. In areas with stained connective cells, there was staining nearby in the basal membranes of rectal gland tubule cells.

**Figure 10:**
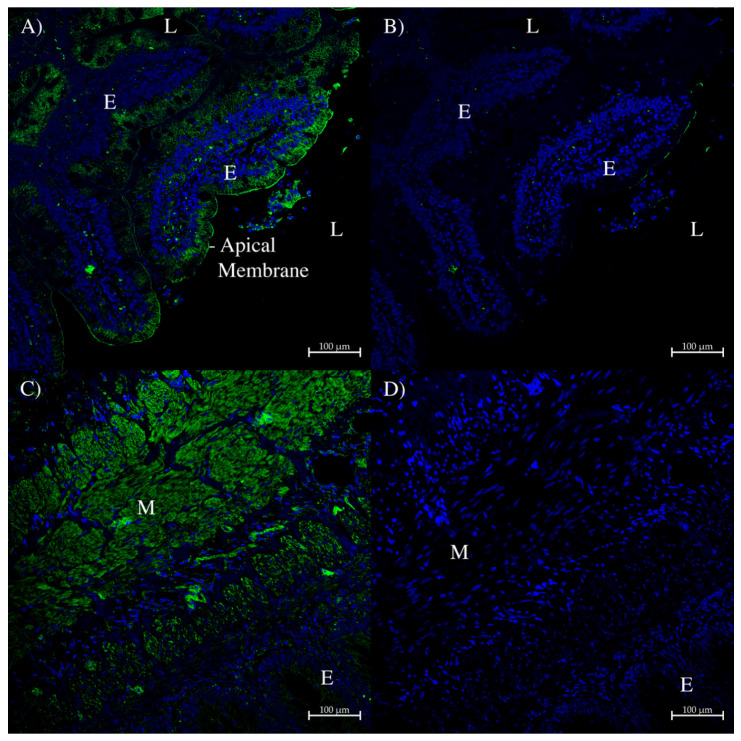
Immunohistochemistry of dogfish intestinal spiral valve side wall using the AQP0 polyclonal antibody. (**A**,**B**) were generated with a tyramide amplification kit using Alexa 488 (green stain; Thermofisher, Carlsbad, CA, USA). (**A**) used the AQP0 antibody, whereas (**B**) was a serial section control image incubated without the primary antibody. (**C**) was incubated with the rabbit AQP0 antibody and an Alexa 488 plus donkey anti-rabbit IgG secondary antibody (green; Thermofisher, Carlsbad, CA, USA). (**D**) was a serial section control image with peptide antigen-blocked AQP0 antibody and an Alexa 488 donkey anti-rabbit IgG secondary antibody (green stain; Thermofisher, Carlsbad, CA, USA). E = epithelium, L = intestinal lumen, M = muscle tissue staining. Images were acquired using a Zeiss LSM 710 laser-scanning confocal microscope (Zeiss, Oberkochen, Germany). All panels were acquired using a 20× lens with a zoom level of 0.6 (scale bars as indicated). All sections had autofluorescence quenched using a True Black plus autofluorescence quencher (Biotium). Blue staining is from DAPI nuclear counterstain present in the Prolong Diamond coverslip mounting medium (Thermofisher, Carlsbad, CA, USA).

In the dogfish spiral valve intestinal side wall, AQP0 staining in the epithelium was very weak and so tyramide (up to 200×) amplification was used to give stronger signals. A small amount of AQP0 staining was seen in the apical and lateral membranes of epithelial cells, especially those in direct contact with the intestinal lumen (Figure 10A). There was also staining in different types of muscle tissue (Figure 10C). One thing to note is that any expression in the muscle would not show up in PCR or Western blot analyses as the samples for those analyses were derived from epithelial scrapes of the tissue that, hence, did not include the muscle layer. Green fluorescent staining was almost absent in the control sections (Figure 10B,D).

**Figure 11:**
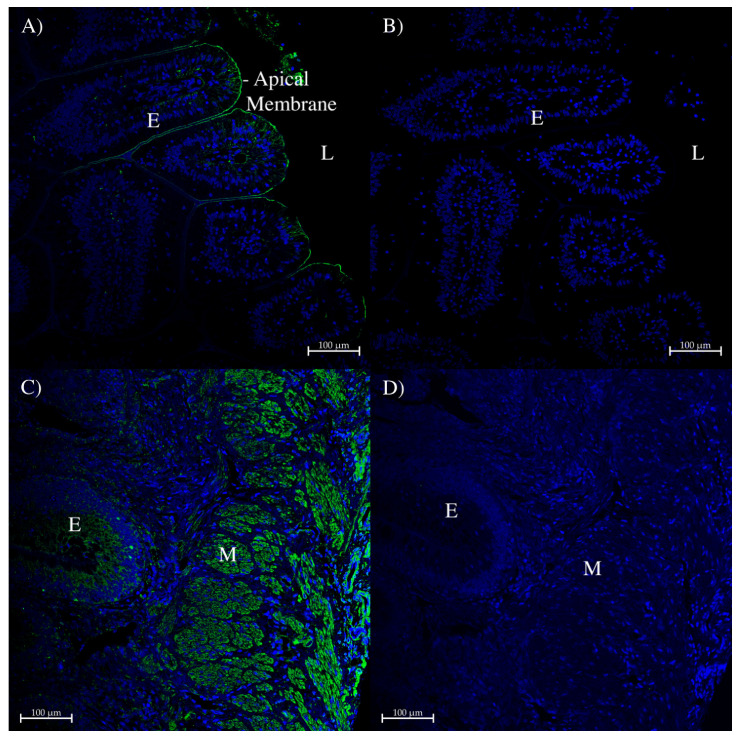
Immunohistochemistry of dogfish intestinal spiral valve flap using the AQP0 polyclonal antibody. (**A**,**B**) were generated with a tyramide amplification kit using Alexa 488 (green stain; Thermofisher, Carlsbad, CA, USA). (**A**) used the AQP0 antibody, whereas (**B**) was a serial section control image incubated without the primary antibody. (**C**) was incubated with the rabbit AQP0 antibody and an Alexa 488 plus donkey anti-rabbit IgG secondary antibody (green; Thermofisher, Carlsbad, CA, USA). (**D**) was a serial section control image with peptide antigen-blocked AQP0 antibody and an Alexa 488 donkey anti-rabbit IgG secondary antibody (green stain; Thermofisher, Carlsbad, CA, USA). E = epithelium, L = intestinal lumen, M = muscle tissue staining. Images were acquired using a Zeiss LSM 710 laser-scanning confocal microscope (Zeiss, Oberkochen, Germany). All panels were acquired using a 20× lens with a zoom level of 0.6 (scale bars as indicated). All sections had autofluorescence quenched using a True Black plus autofluorescence quencher (Biotium). Blue staining is from DAPI nuclear counterstain present in the Prolong Diamond coverslip mounting medium (Thermofisher, Carlsbad, CA, USA).

In the dogfish intestinal spiral valve flap, staining was similar to, but a bit weaker than that in the intestinal side wall. AQP0 staining was mainly seen in the apical membranes of epithelial cells but with some lateral membrane staining in places (Figure 11A). Again, epithelial staining was strongest near the intestinal lumen. There was also staining in different types of muscle tissue (Figure 11C). Green fluorescent staining was absent on control sections (Figure 11B,D).

**Figure 12:**
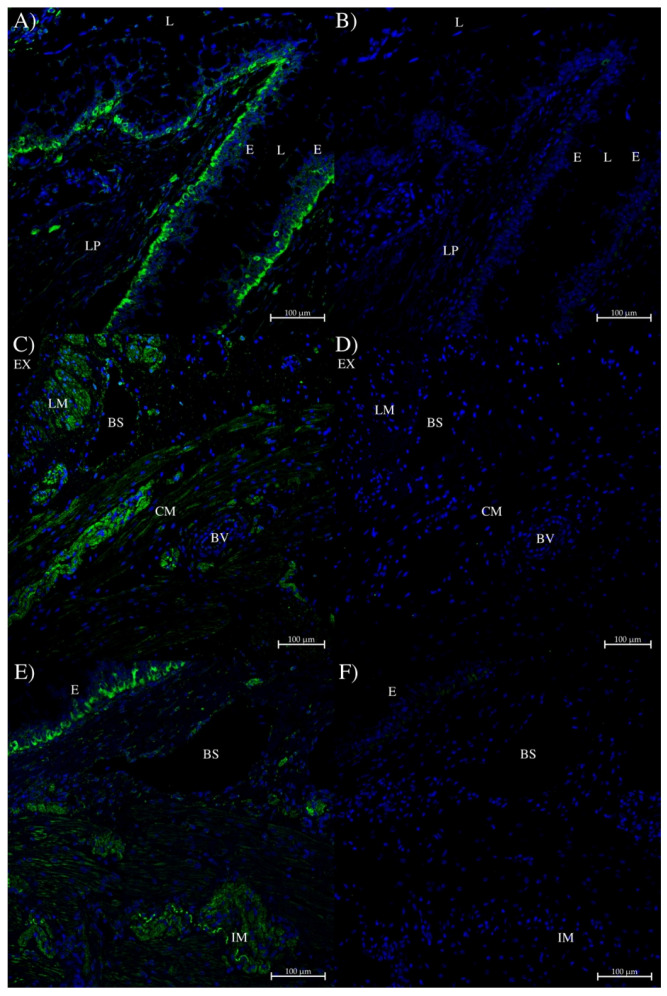
Immunohistochemistry of dogfish intestinal colon/rectum using the AQP0 polyclonal antibody. (**A**,**C**,**E**) were all incubated with the rabbit AQP0 antibody and an Alexa 488 plus donkey anti-rabbit IgG secondary antibody (green; Thermofisher, Carlsbad, CA, USA). (**B**,**D**,**F**) were serial section control images incubated with peptide antigen-blocked AQP0 antibody and an Alexa 488 donkey anti-rabbit IgG secondary antibody (green stain; Thermofisher, Carlsbad, CA, USA) for (**A**,**C**,**E**), respectively. E = epithelium, L = colon/rectal lumen, LP = lamina propria, LM = longitudinal muscle tissue, CM = circumferential muscle tissue, IM irregular muscle staining, BV = blood vessel, BS = blood sinus, Ex = exterior to the tissue. Images were acquired using a Zeiss LSM 710 laser-scanning confocal microscope (Zeiss, Oberkochen, Germany). All panels were acquired using a 20× lens with a zoom level of 0.7 (scale bars as indicated). All sections had autofluorescence quenched using a True Black plus autofluorescence quencher (Biotium). Blue staining is from DAPI nuclear counterstain present in the Prolong Diamond coverslip mounting medium (Thermofisher, Carlsbad, CA, USA).

Dogfish rectal/colon tissue showed strong AQP0 staining in cells that were at the base of the epithelial cell layer (Figure 12A,E). There was also some AQP0 staining apparent at different intensities in muscle tissue (Figure 12C) and an irregular wavy line of staining in some type of muscle tissue (Figure 12E).

**Figure 13:**
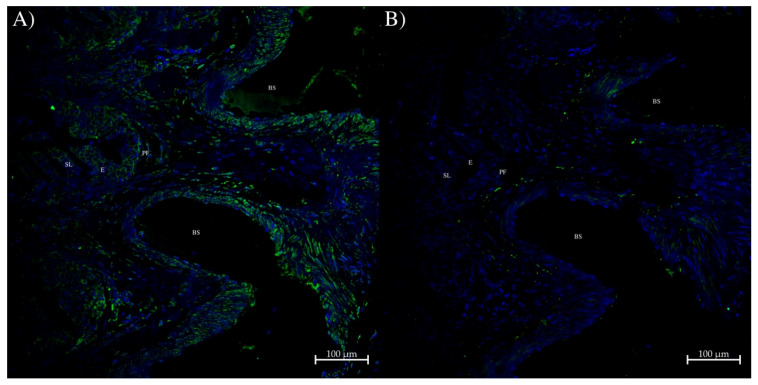
Immunohistochemistry of dogfish intestinal gill using the AQP0 polyclonal antibody. (**A**) was incubated with the rabbit AQP0 antibody and an Alexa 488 plus donkey anti-rabbit IgG secondary antibody (green; Thermofisher, Carlsbad, CA, USA). (**B**) represented a serial section control image incubated with peptide antigen-blocked AQP0 antibody and an Alexa 488 donkey anti-rabbit IgG secondary antibody (green stain; Thermofisher, Carlsbad, CA, USA) for (**A**). E = epithelium, SL = secondary lamellae epithelium, PF = primary filament, BS = blood sinus. Images were acquired using a Zeiss LSM 710 laser-scanning confocal microscope (Zeiss, Oberkochen, Germany). Both panels were acquired using a 20× lens with a zoom level of 0.6 (scale bars as indicated). All sections had autofluorescence quenched using a True Black plus autofluorescence quencher (Biotium). Blue staining is from DAPI nuclear counterstain present in the Prolong Diamond coverslip mounting medium (Thermofisher, Carlsbad, CA, USA).

In the gill, a small amount of AQP0 staining occurred in the secondary lamellar epithelial cell membranes at the base of the primary filament (Figure 13A). There was also some staining in connective tissue surrounding blood sinuses/blood vessels.

## 3. Discussion

The discovery of *AQP0* in spiny dogfish kidney in 2010 was a major surprise as at that point *AQP0* was predominantly thought to be almost exclusive to the lens and was not expected to be found in the kidney. Since then, it has become clear that particularly in teleost fish, *AQP0* expression is not limited to the eye but does also sometimes occur in the kidney and other osmoregulatory tissues [31]. The fact that the AQP0 sequence is highly conserved across a range of shark species suggests that its role is probably relatively important and the fact that this study shows expression of AQP0 in what appears to be renal nephron apical membranes suggests that it is most likely functioning as a water channel in the dogfish in a similar fashion to teleost fish.

The fact that the amplification initially performed using IEJ primers to amplify *AQP0* exon 2 was probably due partly to genomic DNA amplification, but this was unexpected. However, overall further PCR experiments here (Figure 2, Figure 3, and Figure 5) demonstrated that there is at least some kind of *AQP0* transcript expression in all ten of the tissues studied. PCR amplification of cDNA occurred consistently in the kidney, brain, muscle, and eye. Amplification in other tissues was sporadic, and it is currently unclear whether full-length transcripts are present in the pyloric stomach. All parts of the coding region were amplified in different PCR experiments in the gill (overlapping 5′ half and 3′ Race fragments). What illustrates the situation best is the esophagus, where only the full-length coding region was amplified (Figure 2A), but nothing much was amplified in the other PCR reactions. What this all suggests is that for six tissues (gill, rectal gland, esophagus, stomach, intestine, and liver), *AQP0* mRNA/cDNA expression is around the detection limit of PCR (at least using 1 µL of cDNA, i.e., one template molecule per PCR reaction), and that stochastic variability means that sometimes template amplification is present and sometimes it is not.

The presence of the various *AQP0* spliceoform versions was interesting in that (apart from the versions potentially including intron 1) the alternative splicing was occurring at alternative splice sites within the transcript and not at the usual IEJs. The role of these alternatively spliced transcripts is unknown, but given the relatively low level of amplification of them, it seems likely that they will only play some kind of minor role. It is unknown what the role of the *AQP0* transcripts amplified in Figure 5 (including parts or all of intron 1) is. One possibility is that these fragments are partially processed products that are produced during splicing (the removal of introns). However, the lack of a version in muscle cDNA argues against this as it expresses *AQP0* mRNA/cDNA and should possess any processing intermediate *AQP0* cDNAs/RNAs. Intron 1 may be providing an alternative 5′ end to *AQP0* transcripts, but it seems to provide no alternative start codon for normal AQP0 protein translation as there is an in-frame stop codon (TAG) right at the end of the intron 1 sequence. It was also curious that despite the original intestinal RACE product identifying this putative transcript, no amplification from cDNA was obtained from the intestine (of the larger version amplified from cDNA in Figure 5). This suggests that the 5′ RACE product from the intestine may have been amplified from genomic DNA.

For the quantitative PCR, the differences in the means between different experimental groups were not statistically significant. However, the general trend of the data (*AQP0* mRNA/cDNA expression higher in 75% SW fish) was most reminiscent of that of *AQP3-2* mRNA expression, which also had higher (not statistically significant) levels of kidney expression in the same 75% SW RNA samples [43]. In the kidney, *AQP15* mRNA expression was significantly lower in 120% SW dogfish than 75% or 100% SW fish [44], whereas *AQP1*, 3, and *4* expression was higher in 120% SW fish than in the other environments [40,42,44]. Whether these correlations (or contrasting results for that matter) represent meaningful connections (such as *AQP0* and *AQP3-2* expression being co-regulated) is not possible to determine from this study as any connections may also merely be due to random variations in the data.

The Western blot with the custom AQP0 affinity-purified polyclonal antibody showed a band (30 kDa) of around the correct size (28.4 kDa) for AQP0, particularly if the potential unorthodox N-glycosylation site was used, which would add up to 3 kDa to the molecular weight of the protein. The basis of the higher-molecular-weight bands on the gel is unknown. They could be some kind of multimers, as AQP0 is known to multimerize [11,12,13], and the smaller size of the bands on the Western blot (compared to the expected size of 57, 86, or 113 kDa for AQP0 multimers) may be due to N- and/or C-terminal cleavage, as shown to occur in other species [11,12,13,17,18]. Of course, if too much was cleaved from the C-terminal end of AQP0, the antibody would lose its antibody binding site. As ever with polyclonal antibodies, the bands may also be due to cross-reaction to other non-AQP0 proteins. The peptide antibody control blots and immunohistochemistry control sections did at least show that the signals were specific to the antibody.

For the immunohistochemistry studies, in the kidney, the localization of AQP0 in the apical membrane area of the two sinus zone loops (IS I/PII and LDT loops) is similar to that of AQP1 staining in dogfish kidney [42]. Together with AQP1 and AQP15 (at the start of the LDT), AQP0 would presumably provide an influx pathway for water movement out of the nephron lumen. The water would then exit through AQP4 (in the IS I/PII loop) and AQP4 and AQP3-2 (in the LDT loop), which are apparently present in the tubule cell basolateral membranes [41,43]. The LDT in particular is thought to be the main part of the shark nephron where water reabsorption from the urine occurs [45]. There was also some apical staining in one of the five tubules in bundle zones (Figure 8E). The width of the tubule suggests that it might be the end of the IS II nephron segment as it widens out to become the wider EDT segment. The significance of this is not clear, but there is no known basolateral aquaporin protein staining in the IS II segment, so it would seem unlikely for AQP0 to be there for the purposes of transepithelial transport. *AQP0* is related to *AQP1* and *AQP15* (*AQP0*, *AQP1*, and *AQP15* are all derived from *AQP4* during evolution [4]). AQP1 and AQP15 are both expressed in the bundle sheath surrounding nephron bundles [41,42]. Despite that evolutionary relationship, there was no sign of any AQP0 protein expression in the bundle zone bundle sheath.

In the rectal gland, the modest amount of staining in a small region of the gland around the area of the duct is consistent with the low and sporadic level of transcripts seen in the PCR experiments. What was interesting was that as well as some apical membrane staining in a few tubules near the duct, there appeared to be staining associated with a more structural cell–cell adhesion function in some connective tissue cells between tubules in a few places (Figure 9C). This would be consistent with AQP0′s main structural/cell–cell adhesion role in the eye in many species [13,18]. In the intestine, a small amount of AQP0 protein was detected in the apical membranes on parts of epithelial folds in contact with the lumen. The level of intestinal epithelial staining required amplification of the signal to detect it, and hence this suggests that AQP0 must be playing an extremely minor role. AQP0’s role in intestinal muscle tissue is unknown, although its expression would not show up in PCR experiments as the total RNA samples used were produced from epithelial tissue scrapes. Consequently, the level of *AQP0* mRNA and protein expression potentially responsible for the AQP0 staining in intestinal muscle is unknown. Muscle tissue seems to show antibody staining quite often, and muscles can also exhibit autofluorescence, and so the staining in muscle tissues should be treated with caution. In the rectum/colon, as in the kidney, AQP0 staining was somewhat reminiscent of that of AQP1 [42] in that a layer of cells at the base of the epithelium were stained with the antibody, although some individual cells in the middle of the epithelium were also stained. As in the intestine, there was also some variable level and sometimes irregular staining in various muscle layers, but the potential function of AQP0 in these tissues is unknown. Lastly, in the gill, there was weak staining in secondary lamellar cells towards the base of the primary filaments, and also in connective tissue surrounding the blood vessels. As with other aquaporins such as AQP1 that localize to the gill epithelium [42], their role would seem to be largely associated with cell volume control as shark plasma is not drastically different to seawater in osmolality, and hence there are not the same reasons to avoid osmotic water flows that often exist in gills of teleost fish.

Despite the clearly specific (to the antibody) staining of the antibody, the low or sporadic level of mRNA expression in tissues such as the rectal gland, intestine, and gill leaves the immunohistochemical staining results in those tissues somewhat uncertain, and hence they should be treated with caution, despite staining often showing up where it might be expected (in apical cell membranes, etc.). On the other hand, the transcripts that are in those tissues were produced for a reason, and so complete or splice variant versions of AQP0 protein may well be present, as shown in the immunohistochemistry.

## 4. Materials and Methods

### 4.1. Fish Samples

The fish used for the RNA samples used to make cDNA for the PCR experiments and quantitative PCR, and the fish used for the Western blot kidney protein sample and immunohistochemistry tissue blocks, were all processed at the Mount Desert Island Biological Laboratory (MDIBL) in Maine (IACUC approval, MDIBL; e.g., A3562-01; 26 June 2007) and/or Georgia Southern University (e.g., I06050; 27 May 2007). The fish were taken from a stock tank where they were held at ambient temperature in local seawater and fed daily with squid pieces. Gastrointestinal and gill samples for RNA and protein were epithelial scrapes (produced in gill using a razor blade, and in gastrointestinal tract using a microscope slide). The details and conditions for the quantitative PCR and the fish acclimation study are as previously documented [40], but the RNA and protein samples and immunohistochemistry blocks have all been used in many previous studies, including, but not limited to [40,41,42,43,44,46].

### 4.2. PCR, DNA Cloning and Sequencing

The PCR was carried out using Phusion DNA polymerase according to the manufacturer’s (New England Biolabs; NEB, Beverly, MA, USA) instructions using their HF buffer. Ordinary PCR reactions were 20 µL in volume, and the primer annealing conditions were calculated using NEB’s primer Tm calculator; 1 µL of cDNA per reaction was used. The exception to this was the set of reactions shown in Figure 2B, where 5 µL of cDNA was used. To prevent too high a concentration of buffer in the mastermix (normally containing 0.4 units of Phusion DNA polymerase, 4 µL of 5X High Fidelity (HF) buffer, and 0.4 µL of 10 mM dNTPs 12.4 µL dH_2_O) used in those reactions (in Figure 2B), half the buffer (2 µL) was added to the bottom of the PCR tubes, as dH_2_O in the mastermix was reduced to 8.4 µL. For electrophoresis, 1.2% or 1.5% agarose gels were run at a constant current of 265 mAmp and used 1X Gel Red nucleic acid stain (Biotium, Fremont, CA, USA) and 2-log ladder DNA marker (NEB). Gels were visualized on a G Box gel documentation system (Syngene, Cambridge, UK).

As used in some previous studies [46], RACE reactions used cDNAs previously produced using a Smarter RACE cDNA synthesis kit (Takara, San Jose, CA, USA). The original RACE reactions (to clone AQP0) used a single round of touchdown PCR (annealing at 72 °C for 5 cycles, 70 °C for 5 cycles, and 68 °C for 25 cycles) and kidney 5′ and 3′ Smarter RACE cDNA, as in the manufacturer’s instructions, except for the use of Phusion DNA polymerase (NEB). Subsequent RACE PCRs (Figure 3) used two rounds of PCR; the first round (R1) was touchdown PCR as above, followed by nested PCR (R2; 35 cycles, annealing 72 °C) using primers internal to the expected products (see Table 1) and seeding the reactions with 0.5 µL of the first-round PCR reactions as a DNA template.

The original *AQP0* degenerate and RACE and tissue PCR DNA fragments were all cloned and sequenced, as were subsequent RACE fragments (as detailed in Figure 3) and the fragment generated in quantitative PCR (Figure 6). The *AQP0* cDNA sequence had a potential unorthodox N-glycosylation site. For further information, see https://research.bidmc.org/ncfg/blog/facts-about-protein-glycosylation#, accessed on 26 January 2026. Cloning was carried out using a pCR4-TOPO TA cloning kit for sequencing (Thermofisher, Carlsbad, CA, USA), as described previously [46]. Sequences were analyzed using free online software tools such as 4peaks software version 1.8 for sequencing chromatogram analysis (nucleobytes.com, accessed on 26 January 2026). Online software was also used for reverse complementation (bioinformatics.org or vectorbuilder.com), translation (expasy.org, accessed on 26 January 2026), and for sequence alignment (Emboss Needle or Clustal Omega; ebi.ac.uk, accessed on 26 January 2026). Conserved IEJs were taken from the zebrafish *AQP0* (*MIPa*) sequence available in the genome browser at ensemble.org, but also crosschecked with the whitespotted bambooshark (*Chiloscyllium plagiosum*) *AQP0* genomic sequence (NC_057752.1; gene ID:122543350; ncbi.nlm.nih.gov (accessed on 26 January 2026)). Quantitative PCR was carried out essentially as in [42]. For this, the IEJ primers used extended across the junctions of exons 2/3 (sense primer) and exons 3/4 (antisense primer) (see Table 1). The RT-test (i.e., using diluted total RNA as a template) conducted to look for genomic DNA amplification was negative. The quantitative PCR was carried out on a Quantstudio6 real-time PCR machine (Applied biosystems, Thermofisher, Carlsbad, CA, USA). The cycling parameters were 32 cycles with 20 secs annealing/extension at 66 °C. A copy of the 115 bp PCR product was purified, quantified, and serially diluted (1 in 10) from 100 million copies per PCR reaction, and this was used as the amplification standard. The statistical testing of the quantitative PCR data used ANOVA with Fisher’s PLSD post hoc testing between groups, as in [46]. *N* = 6 fish/RNA/cDNA samples per group.

### 4.3. Antibody Production and Western Blotting

The translated amino acid sequence of dogfish AQP0 was used to make a synthetic peptide (NH2-CHPPEAEGQQEPRSDPIELK-COOH), which included an additional cysteine amino acid at the amino terminal end for attachment to a carrier protein (Keyhole Limpet Hemocyanin; KLH). The peptide synthesis, attachment to KLH, immunization of two rabbits, and affinity purification of the subsequent polyclonal antisera using the peptide antigen was carried out by Genscript (Piscataway, NJ, USA). The antibody was used in Western blotting essentially as outlined in [40,41,42], with 300 µg of purified kidney plasma membrane protein using a 1 h incubation with the primary (1 in 400 dilution) and secondary (donkey anti-rabbit IgG antibody; 1 in 4000 dilution; Thermofisher, Carlsbad, CA, USA) antibodies at ambient temperature. As described previously [47], blots were pre-blocked with 5% nonfat milk powder solution and incubated at the end of the procedure in NBT/BCIP with suppressor alkaline phosphatase substate solution (Pierce, Thermofisher, Carlsbad, CA, USA). A separate control Western blot using antibody pre-blocked with the peptide antigen used to make the antibody was also produced. The primary antibody blocking was carried out for >1 h using 50 µg/mL of peptide antigen. The protein molecular weight marker (M) used on blots was Precision Plus Kaleidoscope protein markers (Biorad, Santa Rosa, CA, USA).

### 4.4. Immunohistochemistry

Immunohistochemistry was essentially carried out as in [42] using the rabbit AQP0 custom polyclonal antibody produced. Serial sections (5 µm) were cut from previously available paraffin wax imbedded tissue blocks; see [41,42,43]. The sections were floated onto positively charged (Superfrost plus, Thermofisher, Carlsbad, CA, USA) microscope slides on the surface of a warmed (35–37 °C) and de-gassed dH_2_O water bath. Slides were kept (overnight) almost vertical to drain out water from underneath the sections and then dried on a slide drier set at 42.5 °C. Slides were de-waxed by taking them through two lots of Histochoice clearing agent (wax solvent; Amresco, Thermofisher, Carlsbad, CA, USA), followed by a decreasing series of alcohol solutions (100%, 95%, 85%, 70%, and 50%), 5 min in each solution. Slides were permeabilized using a solution of PBS (phosphate-buffered saline) containing 0.05% Tween20, and the aldehyde groups of the paraformaldehyde fixative were neutralized using a PBS solution containing ammonium chloride. Slides were then blocked in Background Buster (Innovex Biosciences, Richmond CA, USA) for 30 min and then in a solution of 1% BSA and 0.1% gelatin in PBS for 10 min. The primary rabbit anti-dogfish AQP0 antibody was incubated for 1 h (1 in 100 dilution) and then the secondary antibody (a highly cross-absorbed goat anti-rabbit IgG antibody with Alexa488 plus dye; 1 in 1000 dilution; Thermofisher, Carlsbad, CA, USA) for 1 h at ambient temperature. Slides were washed four times with PBS after each antibody incubation. Slide sections were then incubated in Trueblack plus autofluorescence quencher (Biotium) followed by three PBS washes. Then, the slides finally had a coverslip mounted using Prolong Diamond mounting medium with DAPI counterstain (Thermofisher, Carlsbad, CA, USA). Some kidney slides were also additionally incubated with a mouse anti-acetylated tubulin primary antibody and a highly cross-absorbed goat anti-mouse IgG secondary antibody labeled with Alexa 555 dye (Thermofisher, Carlsbad, CA, USA which labels cilia almost exclusively in the proximal half of nephrons) and either rabbit anti-dogfish AQP4/2 or AQP3 antibody directly labeled with CF633 dye (Mix-n-stain antibody labeling kit; Biotium). The AQP4/2 antibody labels the start and middle of the LDT tubule segment and the AQP3 antibody labels the middle and end of the LDT tubule segment [41]. The AQP4/2 or AQP3 antibody slides were pre-blocked (1 h) with 10% rabbit serum after the AQP0 antibody and its secondary antibody incubations, but before the AQP4/2 or AQP3 antibodies were added for 1 h. Slides were washed four times with PBS after all antibody steps. All slides were visualized using a Zeiss LSM710 laser-scanning confocal microscope. Control serial sections used primary AQP0 antibody pre-blocked with the peptide antigen used to make the antibody. The antibody blocking was carried out for >1 h using 50 µg/mL peptide antigen. Some kidney and intestinal sections used a tyramide signal amplification kit with Alexa 488 dye for AQP0 antibody staining (Thermofisher, Carlsbad, CA, USA).

## 5. Conclusions

The level of homology between the AQP0 amino acid sequences between different shark species (around 96–97%) is remarkably high when considering the amount of evolutionary time that the different shark species have been separated from each other for, which is around 250 million years. This level of conservation during that time speaks to the importance of the function of AQP0. While dogfish *AQP0* is clearly very highly expressed in the eye, as in other species including humans, the fairly high level of expression in dogfish kidney was unexpected. As the AQP0 immunohistochemical staining occurred in nephron tubule apical membranes, this suggests that AQP0 plays a role as a plasma membrane water channel. The staining in connecting cells in the rectal gland suggests that AQP0 might also play a smaller role as a cell–cell adhesion protein as in other species.

## Figures and Tables

**Table 1:** AQP0 PCR primers used.

Original Race and Tissue PCR
Squalus AQP0 5′R-QPCR Anti CCTGTGTAAT ACAGCCCGAA GAAGTGACC
Squalus AQP0 3′R-QPCR Sen CTCAACACGC TGCATGCTGG AGT
Race PCR (Figure 3).
Squalus AQP0 5′R-QPCR Anti 2 TCCTGTGTAA TACAGCCCGA AGAAGTGACC
Nested Primer
Squal AQP0 5 Race 2 CTCCACAGCT GTAGCCTGGC CA
Squalus AQP0 3′R-QPCR Sen 2 GCTCAACACG CTGCATGCTG GAGT
Nested Primer
Squal AQP0 QPCR senXL GTGGGTCACT TCTTCGGGCT GTATTACACA
AQP0 Full coding region PCR (Figure 2A).
Squal AQP0 CDS sense AGTCTGCTCT TTGGAAGATC GGGATG
Squal AQP0 CDS Anti CTTTCAAGCA CACCTTCAGT AACAACACAG T
AQP0 Exon 3 amplification (Figure 2B).
Squal AQP0 QPCR senXL GTGGGTC ACTTCTTCGG GCTGTATTACACA
Squal AQP0 QPCR antiXL GACCTACCCA GTACACCCAG TGATTTCCA
AQP0 5′ Half (Figure 2C).
Squal AQP0 CDS sense AGTCTGCTCT TTGGAAGATC GGGATG
Squal AQP0 QPCR antiXL GACCTACCCA GTACACCCAG TGATTTCCA
AQP0 3′ Half (Figure 2D).
Squalus AQP0 3′R-QPCR Sen 2 GCTCAACACG CTGCATGCTG GAGT
Squal AQP0 CDS Anti CTTTCAAGCA CACCTTCAGT AACAACACAG T
AQP0 Quantitative PCR (Figure 6).
Squal AQP0 QPCR sen GTGGGTC ACTTCTTCGG GCTGTAT
Squal AQP0 QPCR anti GACCTACCCA GTACACCCAG TGA

## Data Availability

The authors will make data available to anyone upon request. The original contributions presented in this study are included in the article. Further inquiries can be directed to the corresponding author.
